# An effective method to reduce the interplay effects between respiratory motion and a uniform scanning proton beam irradiation for liver tumors: A case study

**DOI:** 10.1002/acm2.12508

**Published:** 2018-12-13

**Authors:** Yuichi Akino, Huanmei Wu, Ryoong‐Jin Oh, Indra J. Das

**Affiliations:** ^1^ Oncology Center Osaka University Hospital Suita Osaka Japan; ^2^ Department of BioHealth Informatics School of Informatics and Computing Indiana University‐Purdue University Indianapolis Indianapolis Indiana USA; ^3^ Miyakojima IGRT Clinic Miyakojima‐ku Osaka Japan; ^4^ Department of Radiation Oncology New York University Langone Medical Center Laura and Isaac Perlmutter Cancer Center New York NY USA

**Keywords:** adaptive therapy, interplay effects, proton therapy, respiratory motion

## Abstract

**Purpose:**

For scanning particle beam therapy, interference between scanning patterns and interfield organ motion may result in suboptimal dose within target volume. In this study, we developed a simple offline correction technique for uniform scanning proton beam (USPB) delivery to compensate for the interplay between scanning patterns and respiratory motion and demonstrate the effectiveness of our technique in treating liver cancer.

**Methods:**

The computed tomography (CT) and respiration data of two patients who had received stereotactic body radiotherapy for hepatocellular carcinoma were used. In the simulation, the relative beam weight delivered to each respiratory phase is calculated for each beam layer after treatment of each fraction. Respiratory phases with beam weights higher than 50% of the largest weight are considered “skipped phases” for the next fraction. For the following fraction, the beam trigger is regulated to prevent beam layers from starting irradiation in skipped phases by extending the interval between each layer. To calculate dose‐volume histogram (DVH), the dose of the target volume at end‐exhale (50% phase) was calculated as the sum of each energy layer, with consideration of displacement due to respiratory motion and relative beam weight delivered per respiratory phase.

**Results:**

For a single fraction, D_1%_, D_99%_, and V_100%_ were 114%, 88%, and 32%, respectively, when 8 Gy/min of dose rate was simulated. Although these parameters were improved with multiple fractions, dosimetric inhomogeneity without motion management remained even at 30 fractions, with V_100%_ 86.9% at 30 fractions. In contrast, the V_100%_ values with adaptation were 96% and 98% at 20 and 30 fractions, respectively. We developed an offline correction technique for USPB therapy to compensate for the interplay effects between respiratory organ motion and USPB beam delivery.

**Conclusions:**

For liver tumor, this adaptive therapy technique showed significant improvement in dose uniformity even with fewer treatment fractions than normal USPB therapy.

## 1 INTRODUCTION

Particle beam therapy has become an important tool in radiation oncology for cancer treatment due to its improved dose distribution compared to photon beam treatments.^1^, ^2^ However, particle beams are generally more sensitive to organ motion than photon beams.^3^, ^4^, ^5^ This sensitivity is more pronounced in medium with inhomogeneity such as lung and bones, where inter‐ or intrafractional organ motion may significantly change the radiological path length (particle range), thus affecting delivered dose distribution.

Due to concerns over neutron dose in proton beam, beam scanning has recently become an attractive choice.^6^, ^7^, ^8^, ^9^ However, scanned beams are more susceptible to the perturbation caused by scanning motion and interfield organ motion that should be considered.^10^, ^11^ This interplay typically results in under‐ or overdosage within the target volume, depending on the motion and scanning pattern. Phillips et al.^12^ showed that dose uniformity depends on the motion amplitude relative to the direction of beam motion and target motion. Lambert et al.^13^ assessed the interplay for two different scan directions in proton beam therapy and concluded that target margins is not effective in compensating for the effects of intrafractional motion in scanned beam therapy. Furthermore, although range‐based internal target volume (ITV) is commonly used, complexity of using ITV for particle therapy has also been reported.^14^

Recently, several studies have investigated rescanning techniques to reduce interplay effects to improve dose homogeneity.^5^, ^9^, ^15^, ^16^ In this study, we developed a simple offline correction technique for uniform scanning proton beam (USPB) delivery to compensate for the interplay between beam scanning and respiratory motion. Here, we demonstrate the effectiveness of this technique for treatment of liver cancer patients.

## 2 MATERIALS AND METHODS

### 2.A Uniform scanning proton beams

The proton beam used in this study is a nearly continuous beam with an initial energy of 208 MeV. The vertical scanning frequency is 144 Hz, whereas the horizontal scanning frequency depends on the field size (i.e., 14.4 Hz for 10 scanning lines). A detailed description of the USPB technique has been described elsewhere.^6^, ^7^ Unlike spot scanning for intensity‐modulated proton therapy (IMPT), the effects of scanning within the iso‐energy layer will be negligible because the scan speed is much faster than the respiratory cycle. To produce a uniform spread‐out Bragg peak (SOBP), a range modulator consisting of binary combination of graphite plates was used to pull‐back the pristine Bragg peak to different ranges. A 0.5‐s interval is required to change the energy layer by switching the range modulator and is accounted for in the calculation. Because beam layers are pulled back in sequence, a time delay occurs with each beam delivery, consequently leading to interplay effects with moving organs.

### 2.B. Patients, respiratory motion, and treatment planning

Under Institutional Review Board (IRB) exemption, the CT and respiration data of two patients who received stereotactic body radiotherapy (SBRT) for hepatocellular carcinoma were used for simulation. In both cases, the planning target volume (PTV) does not include air (i.e., lung volume). Because the density of liver and surrounding soft tissue is uniform, the variation in dose distribution due to respiratory organ motion is relatively small. Table 1 shows the size, region, and maximum motion range of target volume. The gross tumor volume (GTV) was delineated on the four‐dimensional (4D)‐CT images of the 0%, 25%, 50%, and 75% respiratory phases, and the contours were copied onto the free‐breathing CT images. Clinical target volume (CTV) margin was zero. The original PTV for SBRT treatment was generated by adding a margin to the range‐based ITV, which was the merged volume of the GTVs. Respiratory motion of liver tumors were evaluated with orthogonal cine‐MRI images as shown by Akino et al.^17^ Sagittal and coronal images were acquired for 30 s with the same immobilization of treatment to evaluate the motion amplitude of diaphragm and respiration stability. The motion vectors between two continuous images were analyzed using an optical flow estimation algorithm known as the pyramidal Lucas–Kanade method.^18^, ^19^ After cine‐MRI image acquisitions, planning CT and 4D‐CT images were acquired within 15 min. The 4D‐CT images were sorted into eight respiratory phases. The phases of 0% and 50% accommodate the end‐inhalation and end‐exhalation phases, respectively.

**Table 1:** Characteristics of target volumes and their motion

	Patient # 1	Patient # 2
GTV (cm^3^)	2.0	31.4
PTV (cm^3^)	20.6	128.5
Region^a^	S6	S1
Motion amplitude		
AP (mm)	7.4	0.9
LR (mm)	0.6	1.8
SI (mm)	15.7	7.5

GTV, gross tumor volume; PTV, planning target volume; AP, anterior–posterior; LR, left–right; SI, superior–inferior.

a Regions are shown as the Couinaud classification of hepatic segments.

**Figure 1:**
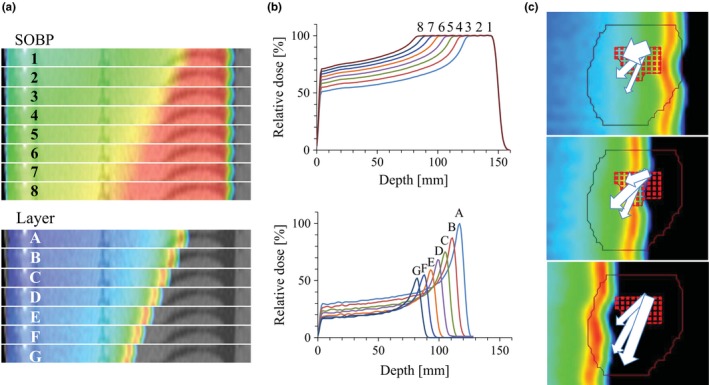
(a) Dose distribution with various spread‐out Bragg peak (SOBP) sizes (upper) and subtracted dose distribution. (b) Depth‐dose profile of SOBP and subtracted layers as illustrated in Fig. 1(a). (c) Scheme of dose‐volume histogram calculation with consideration of interplay effects. Rectangles and line represent segmented voxels of gross tumor volume at 50% respiration phase and planning target volume, respectively. The length and thickness of arrows represent the vectors of respiratory motion and relative weight for the beam delivery of each layer, respectively.

Treatment plans were generated using Eclipse™ treatment planning system version 11 (Varian Medical Systems, Palo Alto, CA). The planning was conducted using the free‐breathing CT images according to the SBRT planning procedure in our clinic. The interplay effects become important, as both organ motion and beam delivery are accompanied with time‐dependent variations with different frequencies. To evaluate the effects properly, three‐dimensional dose distribution of each pristine peak is needed. Unfortunately, Eclipse™ does not provide dosimetric data from each layer in the current version of the software. We therefore considered a rectangle dose distribution that includes the original PTV for the sake of simplicity. We also created multiple rectangular target volumes by varying their thickness in 5‐mm steps and generated treatment plans for each target by fitting the size of SOBP to the rectangular target . The layer‐by‐layer dose distribution data were then obtained by subtracting each dose distribution from larger one [Figs. 1(a) and 1(b)]. The weight of each layer was rescaled to obtain uniform SOBP. The rescaling factor for each layer was determined by checking the profile of summed dose distribution. To simplify the analysis, plans were generated with a single beam at a 0° gantry angle. A 10.0‐cm diameter snout was applied. Treatment fields were collimated with brass apertures. A single set of scanning beams was generated for each patient, and special techniques such as rescanning^15^ were not applied. The treatment time was assessed for the prescribed dose of 2 Gy/fraction.

### 2.C. Offline adaptation of the beam delivery

**Figure 2:**
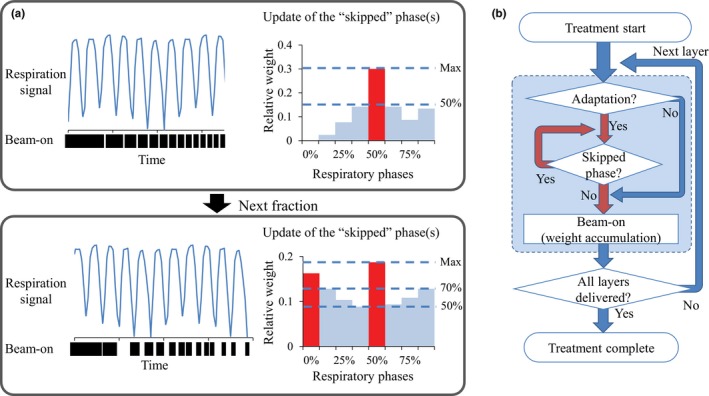
(a) Scheme of offline correction algorithm. The upper and lower side of the respiration signal represent exhale and inhale phases, respectively. Red columns exceeding threshold represent “skipped phase” for the following fraction. (b) Flow chart scheme of offline correction. Red arrows represent adaptation processes.

Figure 2 illustrates our algorithm of offline adaptation for the motion management. Multiple layers of proton beams with various beam‐on times are delivered in sequence. The relative beam weight of each layer among respiratory phases varies based on the beam‐on time and patient respiration. Respiratory‐gated treatment achieves dose distribution similar to simulation by irradiating only at a specific respiration phase. In our method, however, beam delivery is regulated to irradiate uniformly among respiratory phases to achieve uniform dose distribution.

After treatment of each fraction, the relative beam weight delivered to each respiratory phase is calculated for each beam layer [Fig. 2(a)]. The beam‐on time depends on the dose rate. For dose rate of 4 Gy/min, for instance, the beam‐on times of the layer with the smallest beam weight were 1.580 and 772 s for patient #1 and #2, respectively. These layers with short beam‐on time are delivered a part of respiratory cycles, resulting in the inhomogeneous dose distributions. To detect the respiratory phases that received larger amount of beam delivery than other phases, the respiratory phases with beam weights higher than 50% of the largest weight are considered “skipped phases” in the next fraction. For the following fraction, the beam trigger is regulated to prevent beam layers from starting irradiation in skipped phases. If beam delivery starts in a skipped phase, the interval is extended. Although this technique was expected to effectively correct the interplay effects especially for beams with small weight, this may not work for beams with large weights because long beam‐on time results in the uniform distribution of beam delivery among respiratory phases. If the correction successfully achieved uniform distribution of beam delivery after multiple fractions, the correction may not work even for beams with short weight. To overcome this problem, the threshold of 50% was changed to 70% if the beam weight of all phases exceeds 50% of the largest value. If the weights of all phases are higher than 70% of the largest value, adaptation is not applied. In such a case, at the “Adaptation” branched structure in the flowchart, the procedure will go to beam‐on. When all layers have been delivered, the relative beam weights among respiratory phases and skipped phases are updated for subsequent fractions. This algorithm regulates only the interval between beam layers for delivery.

### 2.D. Evaluation of motion interplay effects

To calculate the dose‐volume histogram (DVH), the GTV on the 50% (end‐exhalation) phase of 4D‐CT was evaluated, as patient respiration is most stable in this phase.^20^ Instead of evaluating range‐based ITV, which is a merged volume at each phase, the GTV was moved along the respiratory motion vector, and the average of dose at each position was evaluated for each voxel of GTV. The GTV dose was calculated as the sum of each energy layer with compensation for displacement due to respiratory motion and relative beam weight delivered in each respiratory phase [Fig. 1(c)]. The *Di*, which is the dose delivered to a voxel of target with the 3D coordinates of *Xi,* was calculated using the following formula:

(1) $$D_{i}=\sum_{k}^{n}\sum_{t=1}^{8}\left\{D_{k}\left(X_{i},\delta_{t}\right)\cdot W_{t}\right\}$$

where *D*_*k*_ represents the dose of the pristine peak of the *k*th energy layer; *δ*_*t*_ represents the displacement vector due to the respiratory motion at the respiration phase, *t*; and *W*_*t*_ represents the weight of the beam‐on time for phase *t*. Unlike deformable image registration technique,^21^ this method cannot consider rotation or deformation of tumors for cumulative dose calculation. However, this technique is not accompanied by uncertainties regarding the accuracy of dose warping.

The calculated DVH varies with the initial phase of beam delivery, even if the same respiration data are evaluated. To obtain statistically reliable data, we calculated DVH 500 times. For each iteration, the initial time of starting treatment for each fraction was determined by equally distributed random numbers. To evaluate the total DVH, the dose at each fraction was averaged for each voxel. Therefore, DVH calculation was performed for fraction number multiplied by 500 (i.e., 15 000 times for 30 fractions). The results also vary with the beam‐on time. Under the reference conditions of our system (10 cm cone, energy range of 16 cm, and SOBP of 10 cm), a 2 nA beam current represents a dose rate of almost 2 Gy/min. In the current study, the dose rate for the entire SOBP was assumed to be 2, 4, and 8 Gy/min, and the beam‐on time of each energy layer was calculated by multiplying relative beam weights.

Homogeneity index (HI) defined in International Commission on Radiation Units and Measurements (ICRU) report No. 83^22^ was calculated as follows:

(2) $$\text{HI}=\frac{D_{2\%}-D_{98\%}}{D_{50\%}}$$

where D_2%_, D_50%_, and D_98%_ represent doses of 2%, 50%, and 98% target volume, respectively.

## 3 RESULTS

**Figure 3:**
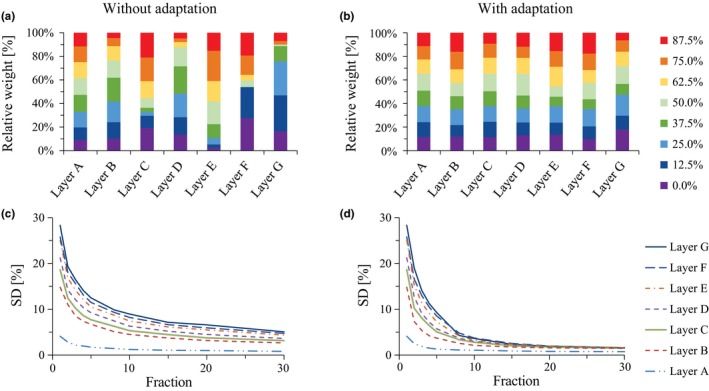
Uniformity of beam delivery among eight respiratory phases calculated for patient #1. An example of relative weight calculated for 10 fractions (a) and (b). Standard deviation of relative weight for each layer was plotted against fraction number (c) and (d).

Figures 3(a), 3(b) illustrate examples of the relative beam weights of beam delivery among each respiratory phase calculated for 10 fractions at a dose rate of 8 Gy/min. Layer 1 with the highest proton energy and longest beam‐on time shows uniform beam weight both with and without adaptation. However, other layers with shorter beam‐on time showed inhomogeneous beam weight without adaptation. In contrast, when adaptation was applied, all layers showed more uniform beam weights than those without adaptation, even for layer G, which had the shortest beam‐on time. In Figures 3(c), 3(d), the standard deviation (SD) of beam weight among each respiratory phase calculated for the 8 Gy/min dose rate was plotted against fraction number, and median values of 500 iterative calculations are illustrated. SD values were larger for layers with short beam‐on time than long. Although data from runs both with and without adaptation showed fraction‐dependent decreases in SD, plans with adaptation showed a much more rapid decrease with increasing fraction number than those without.

**Figure 4:**
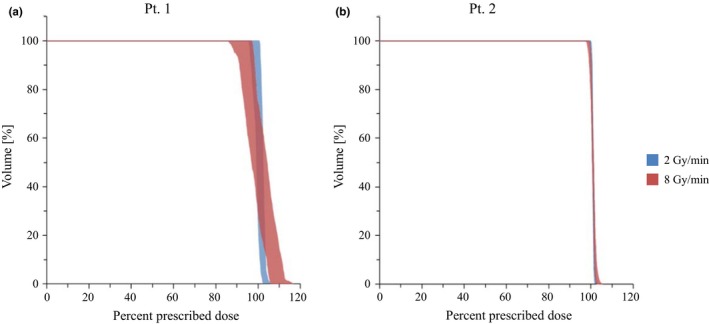
Range of dose‐volume histogram variation calculated for single fraction with dose rates (blue 2 Gy/min and red 8 Gy/min).

Figure 4 shows the range of variation in DVH calculated 500 times for 2 and 8 Gy/min dose rates. The DVH for the 2 Gy/min dose rate was steeper than that for the 8 Gy/min dose rate, which showed large variation with a higher maximum dose and lower minimum dose than the 2 Gy/min dose rate, representing hot and cold spots. In contrast, the histogram of patient #2 showed little difference between 2 and 8 Gy/min dose rates.

**Figure 5:**
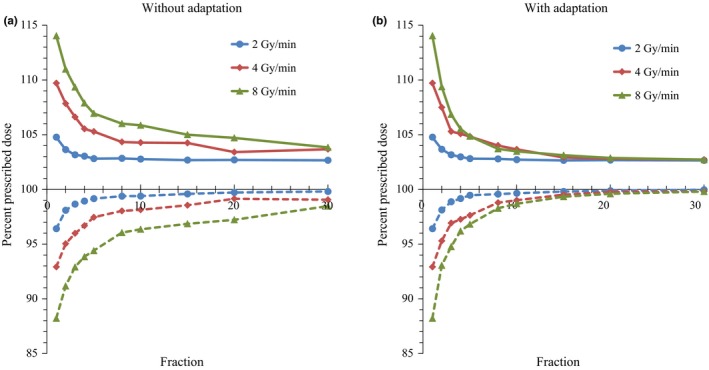
Dose delivered to 1% (solid lines) and 99% (dash lines) of target volume in patient #1 calculated without (a) and with (b) offline correction. The 5% (25th) values from the highest or lowest values of 500 iterations of dose‐volume histogram calculations are shown.

**Figure 6:**
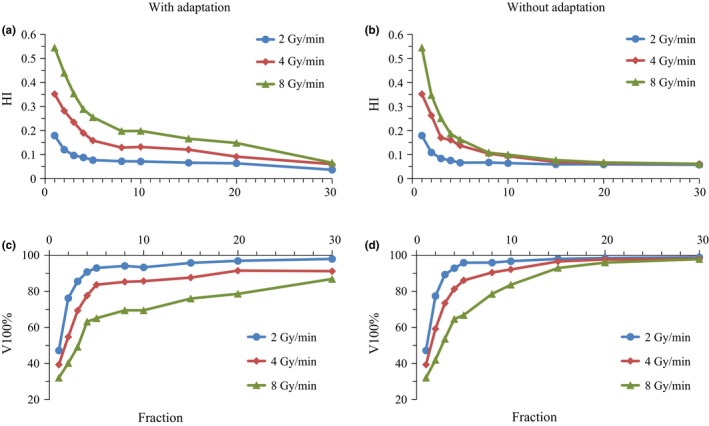
(a) and (b) Homogeneity indices and (c) and (d) relative target volume receiving 100% of the prescribed dose (V_100%_) analyzed for patient #1. Calculations were conducted without (a), (c) and with (b), (d) adaptation of offline correction. The 5% (25th) values from the highest values of 500 iterations of dose‐volume histogram calculations are shown.

Figure 5 illustrates a dose of 1% (D_1%_) and 99% (D_99%_) in patient #1, representing values similar to the maximum and minimum doses, respectively. Figures 6(a), 6(b) shows the HI. The 5th and 95th percentile values, which represent values 25th and 475th from the lowest of 500 iterative DVH calculations, respectively, are shown in Figs. 5 and 6). For single fraction, values 114% of D_1%_ and 88% of D_99%_ were observed for the 8 Gy/min dose rate. Although the D_1%_ and D_99%_ improved with increasing fraction number, inhomogeneity of the 8 Gy/min plan without adaptation remained even at 30 fractions.

In contrast, plans with adaptation showed significant improvement in dose uniformity with increasing number of fractions. Figures 6(c) and 6(d) show the volume receiving 100% of the prescribed dose (V_100%_). For single fraction, V_100%_ of the 8 Gy/min plan was 32%, and while the value improved with increasing number of fractions, V_100%_ was still 86.9% at 30 fractions. In contrast, plans with adaptation showed greater improvement than those without (Figs. 5 and 6), with V_100%_ values in 8 Gy/min plans of 96% and 98% at 20 and 30 fractions, respectively.

Treatment times, including beam‐on time and intervals of plans without adaptation, were 63.5, 33.45, and 18.5 s for 2, 4, and 8 Gy/min dose rate, respectively. Mean ± SD differences in treatment time between plans with and without adaptation were 3.6 ± 1.1, 5.1 ± 1.1, and 4.6 ± 0.8 s for 2, 4, and 8 Gy/min, respectively.

## 4 DISCUSSION

In this study, we simulated the interplay effects between liver tumor motion and beam delivery in USPB. For each fraction, the homogeneity of the delivered dose distribution was poor and correlated with dose rate. Such dose errors in each fraction will be averaged out over multiple fractions, as demonstrated in Figures 5, 6. Although hot and cold spots decreased by averaging dose distribution with multiple fractions, the common 20–30 fractions in a treatment still showed consistent inhomogeneity. We also demonstrated that offline correction of beam delivery by regulating intervals between each energy layer markedly improved dose homogeneity at a lower number of fractions than normal USPB therapy. Although the benefits of adaptation seem modest for a dose rate of 2 Gy/min, improvement in V_100%_ was still observed. Generally the layer with highest energy is delivered with the longest beam‐on time. Although biological uncertainties may remain, due to an inhomogeneous dose being delivered to the target in the initial few fractions, doses at cold or hot spots are not expected to be extremely low or high given the substantial contribution of the beam with the highest energy.

To overcome the effects of organ motion, the gating technique^23^ has been used for moving targets, such as lung^24^, ^25^ and liver^26^ tumors. Based on a signal from a motion‐monitoring device, the beam is delivered only during specific parts of the breathing cycle. Since exhalation is the most reproducible respiratory state, the end‐exhalation phase is often selected as the gating window.^27^ For ion beams including proton and carbon beams, the rescanning technique has been investigated in an effort to reduce dose inhomogeneity.^5^, ^15^, ^16^ In this technique, treatment delivery is repeated *N* times within each fraction, with the number of particles reduced to 1/*N* per rescan. Multiple scans will lead to an averaging effect of the interference pattern if it can be ensured that the motion parameters such as initial phase or respiratory cycle differ for each rescan. Furukawa et al.^28^ showed that the phase‐controlled rescanning method with a large number of rescans improved dose delivery for moving targets. The benefits of the gating technique include minimized interplay effects and potential to reduce field sizes, leading to desired dose delivery to target. However, treatment time increases with gating due to the frequent interruption of the beam. The offline adaptation proposed in this study is simple with active correction, and the difference in treatment time between plans with and without correction is less than 10 s. The baseline shift of patient respiration results in inappropriate beam delivery and often prolongs the treatment time of gating radiotherapy.^29^ In contrast, the baseline shift will not greatly affect our method, especially in terms of treatment time. With this correction technique, uniform dose delivery will be achievable without reducing efficiency of treatment due to elongated treatment time.

For the technique presented in this study, a real‐time monitoring of tumor position is needed. A CyberKnife robotic radiosurgery system (Accuray, Accuray, Inc., Sunnyvale, CA) enables irradiations with real‐time tumor tracking with a Synchrony™ system.^30^, ^31^ The Synchrony system generates a correlation model between the tumor position determined by orthogonal KV x‐ray images and surrogate LED markers placed on the patient chest or abdomen. During treatment, the system keeps monitoring the surrogate LED markers and predicts the tumor position 115 ms ahead of time. Such technology will be helpful for beam regulation with patient respiration. For proton beam therapy, surrogate markers placed on patient chest or abdomen to detect respiration signal may affect the dose distributions. A laser‐based or optical camera‐based devises will be able to provide the signal without affecting the proton beam delivery.^32^ In clinical practice, the range of the ion beam will be affected by density variations, especially in regions including air and ribs. Here, we excluded cases with liver tumors near diaphragm dome and examined patients whose PTV volumes did not include air. For lung cancer treatment, dose calculation will be necessary for each respiratory phase of 4D‐CT. Because the tissue surrounding a liver tumor is solid and the density uniform, the variation in dose distribution due to respiratory motion will be small. However, skin motion due to respiration may also affect the dose distribution, leading to an increase in uncertainties in this study.

With respect to scan direction, several reports have suggested that scanning planes should be perpendicular to the motion direction.^13^, ^33^ In the present study, treatment plans were created using a single beam to generate various sizes of SOBP. Therefore, the dose variation in the lateral and superior–inferior directions is very small, resulting in an underestimation of interplay effects. As shown in Table 1, the motion in the anterior–posterior and lateral direction of patient # 2 is less than 2 mm, resulting in small motion effects. In addition, the target volume of patient #2 is much larger than that of patient #1. In an actual treatment, longer beam‐on time due to a large target volume may lead to modest interplay effects. Generally, two or more beams are used for proton therapy. If two orthogonal beams are used with our methodology, the interplay effects observed in one beam may not appear in another beam because of simple dose distribution. However, if a compensator is applied to the distal edge of a sphere‐shaped target volume, the interplay effects could become more complex and larger, due to the complex dose distribution of each layer. In such cases, motion management may be the choice of treatment to ensure accurate proton therapy.

## 5 CONCLUSION

We have developed an offline correction technique for USPB therapy to compensate for the interplay effects between respiratory organ motion and layer‐by‐layer beam delivery. For the treatment of liver tumors, this adaptive therapy technique showed a significant improvement in dose uniformity with fewer treatment fractions.

## CONFLICT OF INTEREST

This work was supported by a research grant from Varian Medical [No. 4513228].
